# Integrative Radiogenomics Approach for Risk Assessment of Post-Operative Metastasis in Pathological T1 Renal Cell Carcinoma: A Pilot Retrospective Cohort Study

**DOI:** 10.3390/cancers12040866

**Published:** 2020-04-02

**Authors:** Hye Won Lee, Hwan-ho Cho, Je-Gun Joung, Hwang Gyun Jeon, Byong Chang Jeong, Seong Soo Jeon, Hyun Moo Lee, Do-Hyun Nam, Woong-Yang Park, Chan Kyo Kim, Seong Il Seo, Hyunjin Park

**Affiliations:** 1 Department of Hospital Medicine, Yonsei University College of Medicine, Seoul 03722, Korea; nsproper@yuhs.ac; 2 Department of Electronic and Computer Engineering, Sungkyunkwan University, Suwon 16149, Korea; guraud0810@skku.edu; 3 Center for Neuroscience Imaging Research, Institute for Basic Science, Suwon 16149, Korea; 4 Samsung Genome Institute, Samsung Medical Center, Seoul 06351, Korea; jegun.joung@samsung.com (J.-G.J.); woongyang@skku.edu (W.-Y.P.); 5 Departments of Urology, Samsung Medical Center, Sungkyunkwan University School of Medicine, Seoul 06351, Korea; yellowbac@skku.edu (H.G.J.); bcjung2@skku.edu (B.C.J.); ssjeon@skku.edu (S.S.J.); besthml@skku.edu (H.M.L.); 6 Institute for Refractory Cancer Research, Samsung Medical Center, Seoul 06351, Korea; nsnam@skku.edu; 7 Departments of Health Sciences and Technology, Samsung Advanced Institute of Health Science and Technology, Sungkyunkwan University, Seoul 06351, Korea; 8 Department of Neurosurgery, Sungkyunkwan University School of Medicine, Samsung Medical Center, Seoul 06531, Korea; 9 Department of Molecular Cell Biology, Sungkyunkwan University School of Medicine, Suwon 16419, Korea; 10 Department of Radiology and Center for Imaging Science, Samsung Medical Center, Sungkyunkwan University School of Medicine, Seoul 06531, Korea; 11 School of Electronic and Electrical Engineering, Sungkyunkwan University, Suwon 16149, Korea

**Keywords:** renal cell carcinoma, metastasis, radiogenomics, computed tomography, gene expression profiling, imaging surrogate marker

## Abstract

Despite the increasing incidence of pathological stage T1 renal cell carcinoma (pT1 RCC), postoperative distant metastases develop in many surgically treated patients, causing death in certain cases. Therefore, this study aimed to create a radiomics model using imaging features from multiphase computed tomography (CT) to more accurately predict the postoperative metastasis of pT1 RCC and further investigate the possible link between radiomics parameters and gene expression profiles generated by whole transcriptome sequencing (WTS). Four radiomic features, including the minimum value of a histogram feature from inner regions of interest (ROIs) (INNER_Min_hist), the histogram of the energy feature from outer ROIs (OUTER_Energy_Hist), the maximum probability of gray-level co-occurrence matrix (GLCM) feature from inner ROIs (INNER_MaxProb_GLCM), and the ratio of voxels under 80 Hounsfield units (Hus) in the nephrographic phase of postcontrast CT (Under80HURatio), were detected to predict the postsurgical metastasis of patients with pathological stage T1 RCC, and the clinical outcomes of patients could be successfully stratified based on their radiomic risk scores. Furthermore, we identified heterogenous-trait-associated gene signatures correlated with these four radiomic features, which captured clinically relevant molecular pathways, tumor immune microenvironment, and potential treatment strategies. Our results of accurate surrogates using radiogenomics could lead to additional benefit from adjuvant therapy or postsurgical metastases in pT1 RCC.

## 1. Introduction

Renal cell carcinoma (RCC) originates from the highly heterogeneous renal tubular epithelium, resulting in significant inter- and intratumoral heterogeneity in tumor metastasis and therapeutic responses [1,2,3]. In recent years, due to the widespread use of multiparametric imaging, most RCCs have been detected at pathological T1 (pT1) (75%) with a 97% 5-year survival rate, suggesting that most localized RCCs can be surgically treated [1]. Currently, the evaluation of patients for postnephrectomy adjuvant therapy relies mainly on the tumor, nodes, and metastasis (TNM) staging system [3]. However, these RCC subsets exhibit markedly different tumor aggressiveness and diverse clinical outcomes [1,4]. For example, 30% of patients with organ-confined RCC at the time of surgery subsequently recur and metastasize, and they have a universally unfavorable prognosis due to treatment failure (5-year survival rate of 12%) [1,4].

Recently, radiomics has emerged as a powerful tool to comprehensively extract qualitative or quantitative (computer-extracted) phenotypic imaging features from conventional imaging modalities such as computed tomography (CT), magnetic resonance imaging (MRI), and positron emission tomography (PET) [5,6,7]. Radiomics has several advantages, including noninvasiveness, ease of use for serial monitoring, clinical implementation using standard-of-care imaging, and data acquisition from the entire heterogeneous tumor [5,8,9]. Radiogenomics can integrate multiscale data at the fine-grained genome level to more macro-multiparametric imaging data through high-throughput computing to develop new tools to provide insight into relationships between imaging and cellular and subcellular data, reflecting underlying multifaceted and heterogeneous phenotypes [5,6,7,10,11].

RCC frequently exhibits varying amounts of intratumoral heterogeneity, which is exhibited on different spatial scales, such as radiological, macroscopic, cellular, and molecular levels [11,12,13]. RCC is particularly suitable for radiogenomic analysis, as a relative paucity of mutated genes (*VHL*, *PBRM1*, *BAP1*, *SETD2*, and *KDM5C*) allows for more straightforward genomic imaging associations [6,7,11,14,15,16]. Recently, radiogenomics has been focused on elucidating novel imaging-genomic correlates for predicting clinical outcomes and tailored treatment strategies in RCC showing high inter- and intratumoral genomic and molecular heterogeneity [11,12,13]. Especially, postoperative distant metastases develop in a significant proportion of patients designated as cancer-free after curative surgery for localized RCC, primarily due to occult micrometastasis in 20%–35% of patients [17,18]. Therefore, the development of an accurate system to predict patient metastasis is needed to allow for better patient selection of those most likely to benefit from adjuvant therapy. However, to the best of our knowledge, an interpretable and accurate radiomics model using all relevant features associated with RCCs has yet to be reported, and the biological basis of such imaging features has not been fully assessed. In the present study, training and validation cohorts were used to build a radiomic signature, assessing its prognostic value in patients with stage I RCC to predict postoperative metastasis and identify multiscale pathways from genome to imaging levels using a comprehensive radiogenomics approach.

## 2. Results

### 2.1. Selected Prognostic Radiomics Features from the Discovery Cohort

**Figure 1:**
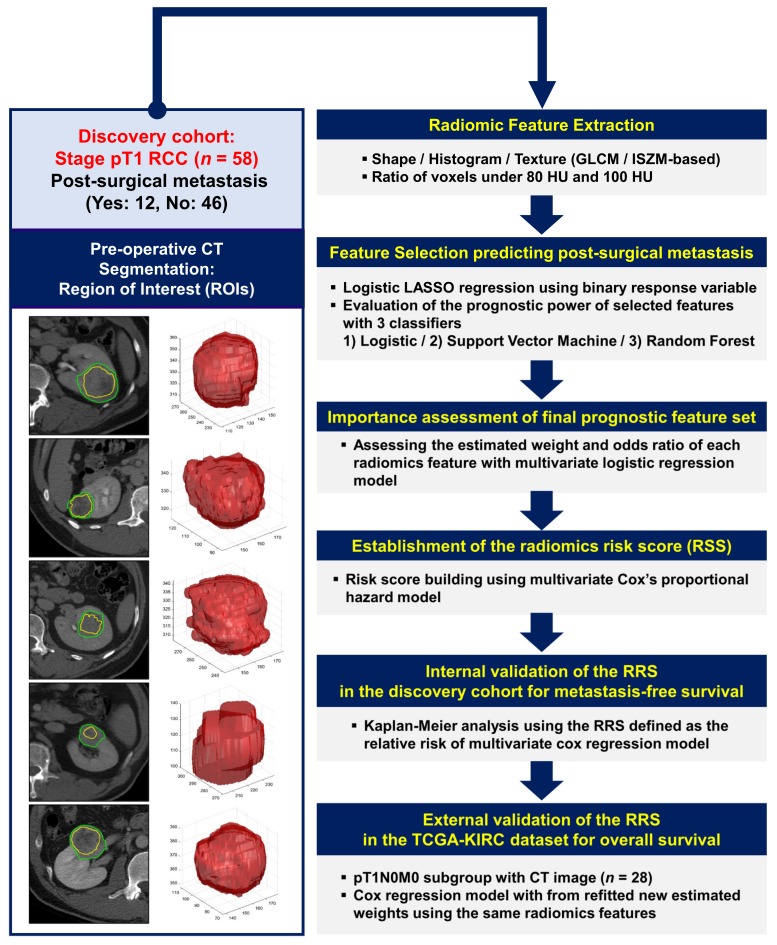
Workflow of radiomics analysis. Manual delineation of representative tumor regions of interest (ROIs) using preoperative computed tomography (CT) (left panel). Radiomic features based on the first- and second-order statistics and two semantic features were computed using inner and outer ROIs. Features associated with postsurgical metastasis in pathologic stage I renal cell carcinoma were selected using the logistic least absolute shrinkage and selection operator (LASSO), and their prognostic values were evaluated using three classifiers (right panel).

The entire radiomics study design is shown in Figure 1. Four radiomics features were selected (noted as radiomics signatures) to predict the postsurgical metastasis of patients with pT1 RCC from the discovery cohort (Table S1). They were the minimum value of the histogram feature from inner regions of interest (ROIs) (INNER_Min_hist), the energy of the histogram feature from outer ROIs (OUTER_Engery_Hist), the maximum probability of the gray-level cooccurrence matrix feature (GLCM) from inner ROIs (INNER_MaxProb_GLCM), and the ratio of voxels under 80 Hounsfield units (HUs) in the nephrographic phase of postcontrast abdomen and pelvis CT (Under80HURatio).

**Figure 2:**
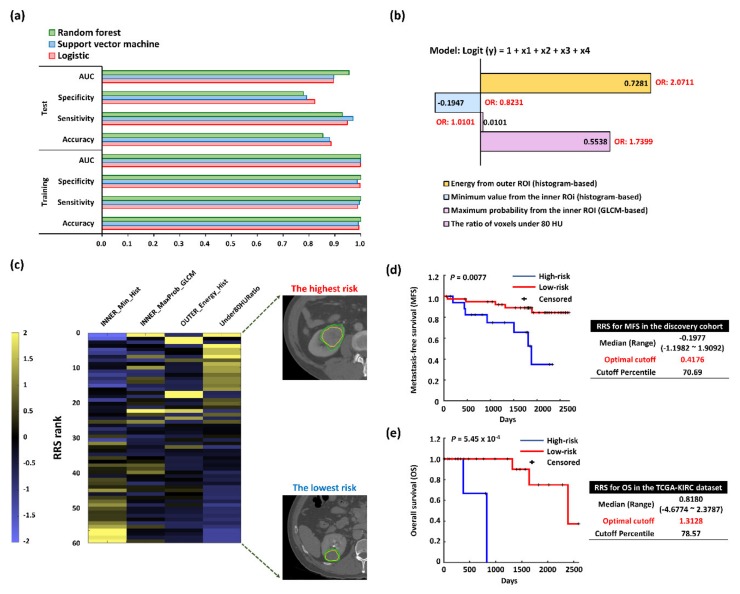
Performance evaluation of the selected features and risk assessment-based radiomics for postsurgical metastasis in pathologic stage T1 renal cell carcinoma. (**a**) Classification performance measures for the selected features. Each performance value was an average of 10,000 bootstrap samples. (**b**) Importance of each finally selected feature using multivariate logistic regression. GLCM, gray-level cooccurrence matrix; ROI, region of interest. Four features were selected to build the radiomics signature using the LASSO logic regression model, and the radiomics score for each patient was calculated. INNER_Min_hist, the minimum value of inner ROIs (histogram-based); OUTER_Energy_Hist, energy feature from outer ROIs (histogram-based); INNER_MaxProbability_GLCM, the maximum probability of the GLCM feature from inner ROIs; (GLCM-based); OUTER_Energy_Hist, the energy of the histogram feature from outer ROIs (histogram-based); Under80HURatio, the ratio of voxels under 80 Hounsfield units (HUs) in the nephrographic phase of contrast-enhanced CT. (**c**) Heatmap showing feature values for patients sorted in descending order by radiomics scores. Each feature value was clipped in the range of –2 to 2 for visualization. High risk scores are in yellow and the associated features are clustered towards the top, while low risk scores are in blue and the associated features are clustered towards the bottom. Two most extreme CT cases are represented at the bottom of the heatmap. (**d**) Kaplan–Meier curves of metastasis-free survival (MFS) rates for high- and low-risk groups according to the radiomics risk score (RRS) in the discovery cohort (*n* = 58). (**e**) Kaplan–Meier plots of overall survival (OS) rates for high- and low-risk groups according to the RRS in the Cancer Genome Atlas Kidney Renal Clear Cell Carcinoma (TCGA-KIRC) dataset (validation cohort; *n* = 28).

The features were selected through the least absolute shrinkage and selection operator (LASSO) based on the logistic regression model. The four features were selected from 9754, 9994, 9536, and 9909 out of 10,000 bootstrapping samples with a 0.2 holdout ratio, respectively. The selected features of each bootstrapped sample were used to train and test three classifiers (logistic regression, support vector machines (SVMs) with a linear kernel, and random forest (RF) classifiers using 50 decision trees [8,9,19]) to distinguish between postoperative metastasis and nonmetastasis (metastasis-free) cases (Figure 2a).

The area under the curve (AUC), sensitivity, and specificity of the RF classifier were all 1 in the training step (accuracy: 1) on average, while the AUC for the test step was 0.9552 with a sensitivity of 0.9288 and a specificity of 0.7786 (accuracy: 0.8537) on average. The AUC, sensitivity, and specificity of the logistic classifier were 0.9989, 0.9879, and 0.9974, respectively, in the training step (accuracy: 0.9926) on average, while the AUC for the test step was 0.894 with a sensitivity of 0.9484 and specificity of 0.8223 (accuracy: 0.8854) on average. The average AUC, sensitivity, and specificity of the SVM were 0.9991, 0.9945, and 0.9867, respectively, in the training set (accuracy: 0.9907), while their respective values were 0.8954, 0.9701, and 0.7902 (accuracy: 0.8801) in the test set.

The multivariate logistic regression was used to assess the importance of the identified radiomic signature for predicting postoperative metastasis as demonstrated in Figure 2b. Three features, INNER_MaxProb_GLCM (0.0101, odds ratio (OR): 1.0101), OUTER_Engery_Hist (0.7281, OR: 2.0711), and Under80HURatio (0.5538, OR: 1.7399), showed positive weights, while INNER_Min_hist showed negative weights towards postsurgical metastasis (−0.1947, OR: 0.8231). The negative weight indicated that smaller values of radiomics features predicted a higher chance of metastasis occurrence.

### 2.2. Radiomics Risk Score (RRS) Predicting Postsurgical Metastasis

The multivariate Cox regression led to the following model for calculating the RRS of each case:

(1) $$\begin{array}{ll} h\left(X_{i},t\right)=h_{0}\left(t\right)\cdot & \exp[(-0.1020\cdot \mathrm{INNER}\_\mathrm{Min}\_{\mathrm{Hist}}_{i}+0.0343\\ & \cdot \mathrm{INNER}\_\mathrm{MaxProbability}\_{\mathrm{GLCM}}_{i}+0.4302\cdot \mathrm{OUTER}\_\mathrm{Energy}\_{\mathrm{Hist}}_{i}\\ & +0.6369\cdot \mathrm{Under}80{\mathrm{HURatio}}_{i})] \end{array}$$

(2) $$\begin{array}{ll} RRS=-0.1020\cdot & INNER\_Min\_Hist_{i}+0.0343\cdot INNER\_MaxProbability\_GLCM_{i}\\ & +0.4302\cdot OUTER\_Energy\_Hist_{i}+0.6369\cdot Under80HURatio_{i} \end{array}$$

where $h\left(X_{i},t\right)$ is the Cox’s proportional hazard of patient i at the time t and $h_{0}\left(t\right)$ is the baseline hazard at time t.

A color-coded heat map was constructed to visualize relationships among the ranges of different individual trait score values, their corresponding composites RRS values, and the binary RRS (high/low RRSs) (Figure 2c). To build a prognostic radiomics model, the analysis was divided into discovery and validation phases (Figure 2d,e). In the discovery cohort, the RRSs of metastasis-free survival (MFS) ranged from –1.1982 to 1.9092 (median: −0.1977), with the optimal cutoff of 0.4176 (70.69%, hazard ratio (HR) = 8.2954, 95% confidence interval (CI) = 2.0957–32.8362, *p* = 0.0077). In the validation cohort (CT imaging data from the Cancer Genome Atlas Kidney Renal Clear Cell Carcinoma (TCGA-KIRC) dataset, *n* = 28, Table S2) [20,21], the RRSs of OS ranged from –4.6774 to 2.3787 (median: 0.8180), with the optimal cutoff of 1.3128 (78.57%, HR = 2.2264 × 10^5^, 95% CI = 1.3878 × 10^3^–3.5719 × 10^7^, *p* = 0.0005).

### 2.3. Functional Enrichment and Prognostic Assessment of Trait-Associated Gene Sets

**Figure 3:**
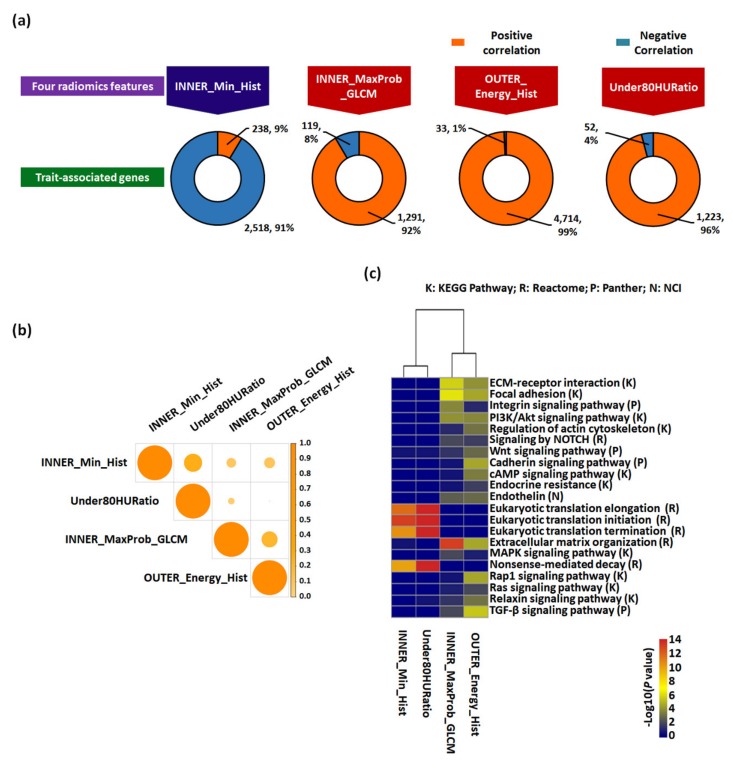
Trait-associated genes and their functional enrichment analysis. (**a**) Each gene set correlated with four radiomic features in the genomic discovery cohort (*n* = 11). Genes that were significantly associated with four radiomic features were identified by Spearman’s correlation analysis, of which significantly correlated genes (*p* < 0.05) were selected. Positive and negative Spearman’s rank correlation coefficients were distinguished by different colors. (**b**) Heatmap of the similarity between each trait-associated gene calculated by the Jaccard similarity index (coefficient). (**c**) Heatmap of pathway enrichment of trait-associated genes. Multiple pathway analyses, including Kyoto Encyclopedia of Genes and Genomes (KEGG) Pathway, Reactome, Panther, and National Cancer Institute-Pathway Interaction Database (NCI-PID), were utilized.

Although only 11 samples among our radiomics discovery cohort were used in the genomic discovery cohort due to the difficulty in obtaining frozen samples that had adequate quality and quantity for the whole transcriptome sequencing (WTS), we further explored the molecular underpinning of the identified all-relevant features by evaluating their possible radiogenomics link using the RNA-Seq technology. To assess the values of radiomics features to capture molecular and biological phenotypic differences of tumors, we curated trait-associated genes correlated to four radiomics features identified in the radiomics analysis (Figure 3a and Table S3).

More than 90% of significant genes correlated to INNER_Maxprob_GLCM, OUTER_Energy_HIST, and Uncer80HURatio had positive correlation coefficients, while 91% of significant genes correlated with INNER_Min_Hist had negative correlation coefficients, confirming the positive and negative estimated weights of the fitted logistic regression model. When we indirectly examined the degree of overlap between radiomics feature-correlated gene sets to investigate the dependence between each feature, all pairs among sets showed an overall low similarity (Figure 3b), suggesting the trait-associated gene heterogeneity in our cohort and the validation cohorts. Among them, the similarities between the gene sets of INNER_Min_Hist and Uncer80HURatio (Jaccard similarity: 26.6%) and between INNER_Maxprobability_GLCM and OUTER_Energy_HIST (20.7%) were the highest, indicating distinct molecular characteristics associated with curated radiomics features.

**Figure 4:**
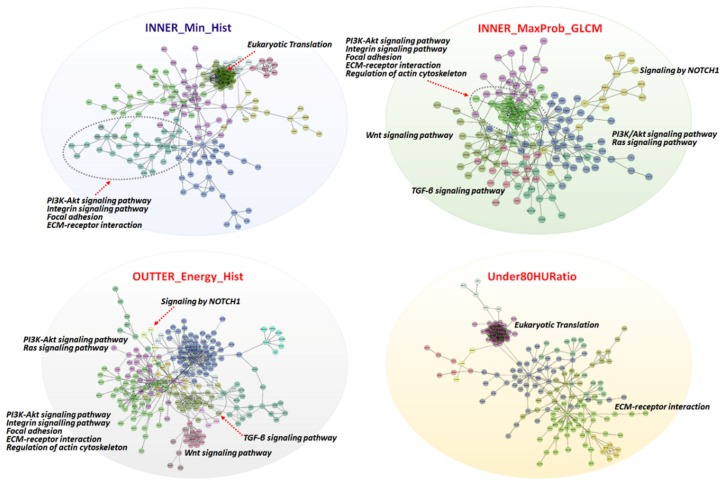
Gene–gene interaction network analysis of trait-associated genes. Gene–gene interaction networks visualized for four radiomic features. Functional gene modules are shown in different colors, and major pathways are annotated.

**Figure 5:**
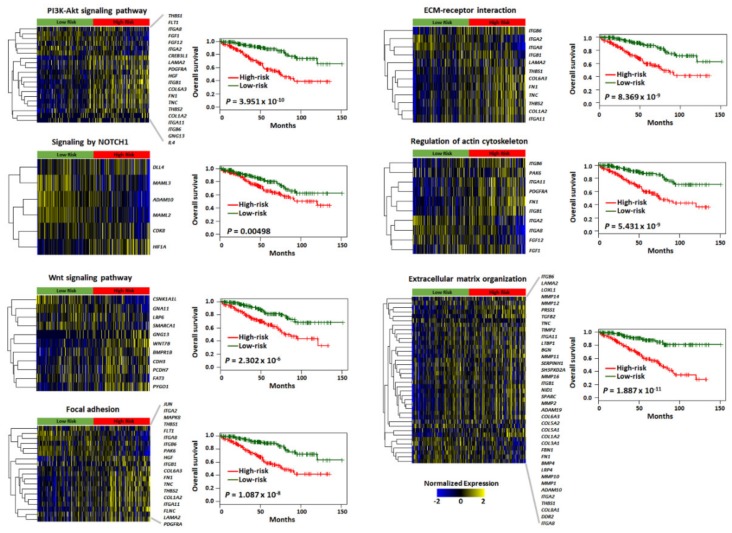
Prognostic significance of major signaling pathways associated with four radiomic features in the genomic validation cohort. The heatmap and Kaplan–Meier curves showing each gene signature score and overall survival difference for high- and low-risk groups via the PI3K/Akt signaling pathway, NOTCH1 signaling, Wnt signaling pathway, focal adhesion, ECM–receptor interaction, regulation of actin cytoskeleton, and extracellular matrix organization.

Molecular functions were annotated of the top-ranked 100 genes in each trait-associated gene set (Table S4) by gene set enrichment analysis, which allowed for the identification of shared common biologic pathways from a public molecular signature database used (Figure 3c and Table S5). Genes associated with INNER_Min_Hist and Under80HURatio were highly enriched in pathways related to eukaryotic translation elongation, initiation, and termination associated with gene translation regulation. Conversely, genes associated with INNER_Maxprobability_GLCM and OUTER_Energy_HIST were enriched in these modules, i.e., “Extracellular matrix (ECM)–receptor interaction”, “ECM organization”, “Focal adhesion”, and “The phosphatidylinositol-3-kinase (PI3K)/AKT signaling pathway”, “Signaling by Notch Receptor 1 (NOTCH1)”, “Wnt signaling pathway”, and “Regulation of actin cytoskeleton”. Similarly, we identified significant functional gene modules by biological network analysis (Figure 4). Notably, the module of “ECM-receptor interaction” was observed in all gene sets of radiomics features. Other modules, including “PI3K/AKT signaling pathway”, “Integrin signaling pathway”, and “Focal adhesion”, were observed in three sets, except Under80HURatio. In particular, “Eukaryotic translation” was modulated in the gene sets of INNER_Min_Hist and Under80HURatio. Collectively, we demonstrated that the four trait-associated genes captured a variety of known molecular classes, suggesting that the radiomics features probed different biologic mechanisms consistent with those shown in Figure 3b. The prognostic values of genes belonging to these significant functional modules (Table S6) were verified in the genomic validation cohort (TCGA-KIRC dataset, *n* = 436, without the M1 stage) (Figure 5).

**Figure 6:**
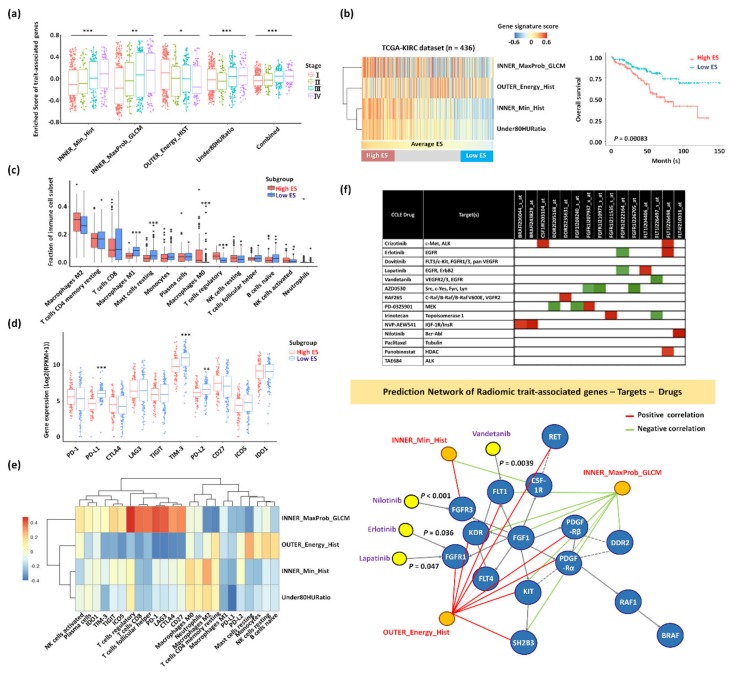
Characterization of subgroups clustered by the expression patterns of trait-associated genes in the genomic validation cohort. (**a**) Comparison of signature scores of each trait-associated gene according to the stage in the TCGA-KIRC dataset (*n* = 436). * *p* < 0.05, ** *p* < 0.01, *** *p* < 0.001. (**b**) Three subgroups (high, intermediate, and low enrichment scores (ESs)) of samples according to the expression patterns of the trait-associated genes in the TCGA-KIRC dataset (*n* = 436) (left panel). Kaplan–Meier curves of OS are shown for high- and low ESs (right panel). (**c**) Differences in immune-cell-type proportions between the high and low enrichment of radiomic gene-sets. (**d**) Differences in checkpoint modulators between the high and low enrichment of radiomic gene-sets. (**e**) Heatmap of the correlation between each trait-associated gene-set and immune-cell-type proportions/checkpoint modulators. (**f**) upper panel: Association between candidate drugs and target genes in kidney cell lines from the Cancer Cell Line Encyclopedia (CCLE) dataset. The correlation map of anticancer drugs and genes belonged to major pathways. The red color indicates a positive correlation between gene expression and the half-maximal effective concentration (EC50, corresponding to less effective drug), and the green color indicates a negative correlation between gene expression and EC50 (corresponding to more effective drug). lower panel: Gene–drug–radiomic feature Network. A directed edge between genes represents activation or catalysis, and a dotted edge indicates the predicted functional interaction.

To investigate the clinical relevance of radiomics features, we clustered the samples according to gene-set enrichment using the RNA-Seq gene expression profile of the genomic validation cohort. The samples were categorized into three subgroups (high, intermediate, and low enrichment scores (ESs)) according to the gene-set ESs of the combined trait-associates genes positively correlated with the stage (Figure 6a,b and Table S7). The high ES group with higher enrichment in the gene signatures of INNER_MaxProbability_GLCM, INNER_Min_Hist and Under80HURatio had an unfavorable prognosis than the low ES group with higher enrichment in the gene signatures of OUTER_Energy_HIST (*p* = 0.00083) (Figure 6b).

### 2.4. Trait-Associated Genes Reflective of the Tumor Immune Microenvironment and the Predicted Drug Sensitivity

To tailor immunotherapeutic treatments for metastatic RCC, the characterization of radiogenomic features measured at diagnosis and throughout treatment may be relevant to grasp changes in gene expression patterns and the tumor immune microenvironment [11]. We conducted a comprehensive and detailed assessment of the association between radiomics features, the infiltration of immune cell types, and the expression patterns of immune-checkpoint molecules (Figure 6c–e and Table S7). We found that the high ES group with poor prognosis had higher fractions of M0 macrophage and regulatory T cells (Tregs) closely associated with INNER_MaxProbability_GLCM, INNER_Energy_HIST, and Under80HURatio. In addition, T-cell-associated cell types, including regulatory, CD8, and follicular helper, and immune checkpoint proteins, including programmed cell death protein 1 (PD-1), lymphocyte-activation gene 3 (LAG-3), and cytotoxic T-lymphocyte–associated antigen 4 (CTLA4), were highly positively correlated with INNER_MaxProbability_GLCM. We found significant differences in proportions of radiomics feature gene sets between high and low ESs in M0 and M1 macrophages, Tregs, and activated natural killer (NK) cells (*p* < 0.05). Among 10 checkpoint modulators, programmed death-ligand 1/2 (PD-L1/PD-L2) and T-cell immunoglobulin and mucin domain-3 (TIM-3) were significantly decreased in high ES cases.

As imaging biomarkers may have clinical implications in aiding patient selection for targeted therapies [5], we examined the association of anticancer drugs and their target genes using RCC cell lines from the Cancer Cell Line Encyclopedia (CCLE) dataset [22]. A number of drug–target relationships (i.e., sensitive or resistant) between gene expression and tumor response were identified (Figure 6f). For example, the high expression level of fibroblast growth factor receptor 1 (*FGFR1)* was more effective against erlotinib, and lapatinib showed a negative correlation (*p* = 0.036). Nilotinib may inhibit *FGFR3* (*p* < 0.001), and vandetanib may target Fms-related tyrosine kinase 1 (*FLT1*) (*p* = 0.0039). The *FGFR1* and *FLT1* target genes were positively correlated with the radiomics feature of OUTER_Energy_HIST. The *FGFR3* target gene was positively correlated with INNER_Min_HIST. Conversely, the *FLT1* target gene was negatively correlated with INNER_MaxProbability_GLCM. Overall, imaging biomarkers and trait-associated genes could suggest specific immunotherapeutics and targeted drugs in pT1 RCC patients with metastasis.

## 3. Discussion

Herein, we curated a radiogenomics model predictive of postsurgical metastasis and their molecular properties in pT1 RCC patients to help physicians select high-risk patients most likely to benefit from early systematic therapy and ultimately be used for the assessment of optimal therapy based on imaging features. To avoid overfitting or bias, we performed a robust statistical validation. In the prognostic feature-discovering step, the RF classifier performed the best among the three classifiers with a mean AUC of 0.9552 in the holdout test of 10,000 bootstrapping trials. The selected radiomics signatures led to a significant group difference of HR of 8.2954 in the MFS analysis. Further, we conducted a survival analysis in a radiomics validation dataset limiting inclusion to only pT1 patients, confirming the results were obtained from the independent cohort. The clinical relevance and biological meaning of novel radiomics signature containing four radiomics features, most of which currently have no known significance, were determined in a discovery cohort and confirmed in an independent open dataset. Our study showed that a radiomics signature is a strong preoperative predictor for the distant metastasis of T1 RCC, which might lead to a better stratification of patients for surgery, enabling clinicians to choose optimal treatment strategies and individualized monitoring protocols to enhance clinical outcomes.

In this retrospective single-center study, a novel radiomics model was built with two histogram-based features, one textural feature, and one semantic feature. In contrast to conventional imaging features from CT, texture analysis based on contrast-enhanced CT imaging could offer objective information about intratumor heterogeneity, and correlating the analysis results with gene-expression patterns might help in predicting clinical outcomes [5,6,7,10,11]. A precise understanding of intertumor heterogeneity may facilitate pertinent the triage of patients to effective therapies, as well as the anticipation and treatment of resistant lesions. Increased intratumor heterogeneity associated with adverse clinical outcomes [10] can be quantified using parameters such as skewness, kurtosis, and entropy [8,9,23,24,25,26]. For example, another study of metastatic RCCs showed that entropy, a CT texture feature reflecting tumor heterogeneity, was an independent factor associated with time to progression [26]. Notably, none of the radiomics features were from the whole tumor ROI, suggesting that partitioning the whole tumor into the core and peripheral regions was effective for prognostic model building compared to using the whole tumor ROI, consistent with previous studies [27,28]. Especially, INNER_MaxProbability_GLCM was closely associated with genes enriched in the high-risk group, reflecting intensity heterogeneity in the tumor core region [29]. A GLCM is a two-dimensional (2D), second-order matrix that measures the frequency of a given gray-scale value at a predefined interval and direction from another [8,9,24]. Additionally, the INNER_Min_HIST represented the darkest intensity in the tumor core region, which could be related to the hypointense necrotic core. Under80HURatio is a semantic feature, which can reflect the proportion of the unenhanced portion of the tumor. The feature could be associated with decreased metabolism in the necrotic portion [29]. All proven RCCs contained substantial noncalcified ROIs measured in 20–70 HUs on unenhanced CT [30]. Hypoxic tissues exhibited greater spread and variation in voxel values on contrast-enhanced CT [31]. Tumor necrosis was considered present, if low-attenuation areas were visually identified with the corresponding HU measurements between 10 and 30 [32] and without any relevant HU increase (15–20) between precontrast and contrast-enhanced CT images [33,34]. Increased nonenhancing tumor and necrosis in RCC have the potential to serve as an imaging-based biomarker predicting an elevated incidence of a higher pathological stage, cancer recurrence after resection, and cancer-specific mortality in RCC [35,36].

Although the metastasis of RCC is predominantly observed in late-stage tumors, early-stage metastasis can be found through an undefined molecular mechanism, leading to inappropriate clinical decisions and poor prognosis. To date, the mechanism of metastasis is poorly understood [37]. Moreover, to achieve biological relevance and decipher mechanisms underlying the classification radiomics model, future studies are necessary to combine individual CT features into CT imaging phenotypes of RCC and correlate them to underlying multiple regulators in cancer pathways rather than individual gene mutations [5,11]. Transcriptomics is of particular interest in oncology for the identification and quantification of RNA in cells, tissues, or biological fluids, representing a powerful tool for the assessment of specific biological activities [19]. The radiogenomic analysis of pT1 RCC showed multiple associations between semantic image features and trait-associated genes that reflected the distinct underlying molecular basis of distant metastasis after surgery, and this system provided important prognostic information and identified high-risk patients, who may benefit from early adjuvant treatment. Looking at the molecular pathway level, the gene ontology analysis revealed associations between imaging groups and gene pathways in cancer oncology [5]. The presented image-to-molecular feature associations could be used to assess therapeutic options based on biologic pathway activity using surrogate image features. Here, we confirmed the prognostic relevance of trait-associated genes in the context of predicting recurrence and survival in early-stage RCC and identified its association with ECM remodeling, focal adhesion, mRNA translation, PI3K/AKT signaling, NOTCH1 pathway, WNT pathway, and cell cytoskeleton, enabling an in-depth understanding of noninvasive RCC biology using CT images and increasing the potential of radiogenomics maps. Hypoxia and necrosis determined as significant radiomic features played a central role in RCC progression by modulating critical signaling pathways including PI3K/AKT signaling, Notch signaling, Wnt signaling, and focal adhesion, governing stem-cell-like phenotype, cancer cell plasticity, metabolic reprogramming, epithelial-to-mesenchymal transition (EMT) closely associated with higher probability to escape the primary tumor and survive in an avascular metastatic site [2,5,21,38,39,40]. The aberrant overexpression of key proteins via cap-dependent mRNA translation is known to be a requisite for tumor progression and metastasis via the strong activation of the eukaryotic initiation factor (eIF) 4E-binding protein 1 (4EBP1/eIF4E) axis in RCC [41]. Furthermore, an ECM remodeling program and an ECM-receptor interaction pathway facilitate cell survival, angiogenesis, EMT, and metastasis of RCC via the subsequent activation of the focal adhesion kinase (FAK)/c-Jun N-terminal kinase pathway and PI3K/AKT pathway [42,43,44]. Finally, cytoskeletal-rearrangement-associated proteins rearrange the actin cytoskeleton driving cell cycle progression, invasiveness, and EMT [45,46,47]. Importantly, we found that FGFR1 and FGFR3 target genes were enriched in OUTER_Energy_HIST- and INNER_Min_HIST-associated genes, and RCC characterized by OUTER_Energy_HIST and INNER_Min_HIST could be effectively targeted by erlotinib, lapatinib, and nilotinib based on drug–target relationships using kidney cell lines from the CCLE dataset [22]. FGFR upregulates epidermal growth factor receptor (EGFR) signaling, and these two signaling (mediated by FGFR1 and FGFR3) control critical orchestrators at the convergence of EMT pathways, such as PI3K/AKT, mitogen-activated protein kinase (MAPK), and Wnt/β-catenin axis [48,49,50,51]. The gene pathway analysis in this study revealed imaging genomic networks in oncology and indicated that radiogenomics may be suitable for predicting the efficacy of pathway-targeted therapies [5].

The tumor microenvironment of RCC is infiltrated by high levels of different immune components, and the composition and function of immune cells usually change during RCC progression [52,53]. Cancer immunotherapy has recently received a great deal of attention again due to the success of immune checkpoint blockade (ICB), which reactivates antitumor effector CD8+ T cell-mediated cancer cell death via the disruption of immune tolerance [52], although the response rate to ICB therapy remains at 20%–30% depending on cancer type [52]. As texture analysis features could be determined by the underlying immune components of the tumor microenvironment at multiple scales [54,55], radiogenomic models can be used to further elucidate the prognostic utility of the local immune environment and can be enhanced to predict or monitor response to immunotherapy agents. In this study, M1 macrophages associated with OUTER_Energy_HIST were enriched in low ES, while M0 macrophages associated with INNER_Energy_HIST and Under80HURatio were enriched in high ES. Especially, we found that the high ES group with poor prognosis had higher fractions of M0 macrophages and Tregs closely associated with INNER_MaxProbability_GLCM, INNER_Energy_HIST, and Under80HURatio. In addition, immune checkpoint proteins on the T-cell surface, including PD-1, LAG3, and CTLA4 [52], correlated with INNER_MaxProb_GLCM-associated genes. Macrophages are the most abundant subpopulations of tumor-infiltrating immune cells, and high M0 and M2 macrophage fractions were related to a poor outcome, which might reflect their gradual change in function in RCC [52,53]. The tumor-promoting effects of EMT shift to M1 type, which are considered to exert anticancer effects via cytotoxicity and immune rejection to M2, similar to tumor-associated macrophages that are considered to promote cancer growth via EMT, angiogenesis, and immunosuppression [52,56]. Tregs combined with CTLA4, LAG3, and TIGIT related to a worse prognosis in RCC exerted immunosuppressive effects, favoring tumor escape from the activity of a variety of antitumor immune effector cells [52,53]. Interestingly, heightened Wnt signaling in the tumor microenvironment (TME) correlates with immunoevasion such as the suppression of T cell infiltration and/or function and alterations in immune checkpoint molecules [57,58].

This study has various strengths and weaknesses including the retrospective design at a single institution with small sample sizes, the limited availability of fresh frozen samples with adequate quantity and quality for genomic analysis and correlative but not causative relations between molecular and imaging phenotypes. The sample size of the discovery cohort is very important for the reliability and the predictive power of the radiogenomics study. However, it is not easy to obtain many pT1 RCC cases diagnosed with distant metastasis after curative nephrectomy due to a low incidence rate. Whole transcriptome sequencing requires high-quality frozen tissue samples, which makes the data collection even more challenging. Future investigation with well-designed prospective multicenter data should be conducted to confirm molecular mechanisms that may drive the imaging behavior.

Complementary innovations in genetic, epigenetic, and proteomic analysis and an imaging-based analysis that allow for the spatial and temporal quantification of tumor heterogeneity and its changes during tumor evolution could provide a basis for the realization of precision oncology. Although we focused on RCC, the strength and breadth of our results are encouraging, and these radiogenomic approaches for biomarkers may have useful future applications for detecting and tracking diseases, with interesting future applications for RCC and potential applications for other diseases.

## 4. Materials and Methods

### 4.1. Patient Cohorts and Image Data Collection

For imaging biomarker discovery, all analyses were approved by the appropriate Institutional Review Board at Samsung Medical Center (Seoul, Korea) (IRB No: 2019-09-005) for a retrospective design and waiver of informed consent regarding the acquisition of CT data. The discovery cohort used to construct the RRS consisted of RCC tumors treated with radical or partial nephrectomy between March 2010 and May 2016 at Samsung Medical Center (Seoul, Korea), fulfilling the following inclusion criteria: (1) histopathologic diagnosis of RCC (58 cases: clear cell subtype), (2) stage I based on pathologic findings following the American Joint Committee on Cancer staging manual, eighth edition: T1N0M0 [59], (3) availability of contrast-enhanced abdomen and pelvis CT scan performed prior to surgery, (4) availability of clinical follow-up data regarding postoperative metastasis events and MFS events, and (5) lack of postadjuvant therapy. In total, 58 patients were included, which consisted of 12 cases with postoperative metastasis in common sites [60] (1 case: adrenal gland, 1 case: adrenal gland and pancreas, 2 cases: pancreas, 8 cases: lung parenchyma) and 46 cases with no evidence of disease (Table S1). MFS was measured from the date of surgery to the date of the first clinical evidence of metastasis and was censored at the date of death or the date of the last follow-up visit for survivors.

Publicly available resources containing multidimensional datasets such as the Cancer Imaging Archive (TCIA) and The Cancer Genome Atlas (TGCA) are especially useful given the hurdle in obtaining genomic data, due to the need for small, fresh tissue samples and for the amount of data that can be extracted for both hypothesis-generating and validation purposes [20,21,61]. Herein, for a radiomics validation cohort, we identified patients in the TCGA-KIRC dataset fulfilling the following inclusion criteria [20,21]: (1) stage I RCC, (2) the availability of preoperative contrast-enhanced CT scan, which had similar image acquisition settings to the discovery cohort (standard of care abdomen and pelvis CT performed before surgery), and (3) the availability of survival state and OS data to validate the survival model derived from the prognostic features using OS information (since patients with recurrence information were limited). The final study population consisted of 28 patients, including 5 patients with death events and 23 censored patients (Table S2).

### 4.2. CT Imaging and Specification of the ROI

All preoperative contrast-enhanced abdomen and pelvis CT examinations were performed using one of multidetector row CT scanners (Brilliance 40, Philips Healthcare, Cleveland, OH, USA; LightSpeed VCT, GE Healthcare, Milwaukee, WI, USA; Somatom Definition FLASH, Siemens Healthcare, Forchheim, Germany) with 40, 64, or 128 detectors. The acquisition scanning parameters were as follows: collimated beam size from the detector, 40 mm × 0.625 mm, 64 mm × 0.625 mm, or 128 mm × 0.6 mm; voltage: 120 kV; current: 180–200 mA; and reconstruction interval: 2.5–5 mm. Multiplanar-reformatted images of the sagittal or coronal plane were obtained from datasets of the nephrographic phase of postcontrast images. Nephrographic phase images were obtained 100 s after the initiation of an intravenous injection of a nonionic low-osmolality contrast material (Iomeron 300, Bracco, Milan, Italy) at a rate of 2.5–3 mL/s via an automated injector. A radiologist (C.K.K., with 15 years of experience in genitourinary imaging) without access to clinical outcomes and genomic data, but aware of the presence and site of a RCC, drew manually the ROIs of the whole tumor on the axial plane for each CT image using MRICro software (https://people.cas.sc.edu/rorden/mricro/mricro.html). A picture archiving and communication system (PACS; PathSpeed Workstation; GE Medical Systems, Milwaukee, WI, USA) was used for image analysis.

We designed a custom library of CT features that captured various aspects of tumor physiologic and morphologic characteristics, tumor microenvironment, and local tumor-parenchyma ecologic characteristics, which would enable the subsequent construction and identification of RCC radiophenotypes. The radiomics features were computed from the ROIs of the previous section for all patients using the open-source software PyRadiomics that enables quantitative imaging annotations [62]. Three types of ROIs were considered: whole, outer, and inner ROIs. The inner and outer ROIs quantified characteristics of the stable tumor core and dynamic peripheral region, respectively. Morphological features from the whole ROI were used to quantify geometrical characteristics of the tumor.

### 4.3. Image Analysis and Texture Feature Extraction

Radiomics features were extracted from the ROIs extracting intensity, shape, size, and texture information [8,9]. Some features were computed using first-, second-, and/or higher-order statistical methods [8,9]. First-order features are generally histogram-based techniques, which reduce an ROI to single-value representation for entropy (a statistical measure of randomness within a data sample), kurtosis (a parameter that depicts the degree of peakedness (broad or narrow) of a histogram), and the maximum, mean, median, minimum, skewness (a parameter that describes the asymmetry of a histogram around the mean), and uniformity of the intensities on a radiological image [8,9]. Second-order statistical descriptors that characterize texture features describe spatial relationships between voxels with similar gray levels within a lesion, providing a measure of intralesional heterogeneity [8,9]. Conventional techniques include the GLCM, intensity size zone matrix (ISZM), gray level run-length matrix (GLRLM), and gray tone difference matrix (GTDM) [8,9]. In this study, histogram-based features were computed using 128 bins to robustly quantify properties related to the intensity distribution. GLCM and ISZM features that quantify intratumoral heterogeneity were computed using 128 and 32 bins, respectively. Initially, 119 radiomic features were extracted from images for each patient to describe tumor characteristics, including 8 morphological features, 19 histogram-based features, and 16 GLCM-based features. Further, two ISZM-based features were extracted for each RCC (i.e., $119=\left(19+16+2\right)\times 3ROIs+8$. In addition, to measure the nonenhancing portion in the tumor, two semantic features, the ratios of voxels under 100 and 80 HUs, were computed by contrast-enhanced CT using an in-house MATLAB code.

### 4.4. Radiomics Model Building and Survival Analysis

The discovery cohort was highly biased towards nonrecurrence cases (46 nonrecurrence and 12 recurrence cases), causing difficulty in the application of machine learning strategies because most machine learning algorithms are designed to be effective on balanced data. To overcome this limitation, we applied the adaptive synthetic (ADASYN) oversampling algorithm [63] to the discovery cohort and obtained an equal number of nonrecurrence and recurrence cases (47 for both cases in our cohort). Feature selection can be performed using classification or regression procedures, identifying a subset of features that can be used to train highly accurate predictors of individualized outcomes [8,9]. As an embedded feature selection, we employed the LASSO regularization [64] within logistic regression using R software (R Foundation for Statistical Computing, Austria) [65]. This method is useful in regression analysis for high-dimensional data, and it can combine a set of selected features into a radiomics signature [65]. The binary state of metastasis (no metastasis/metastasis = 0/1) was used as a response variable. The logistic LASSO procedure was performed with five-fold cross-validation and repeated 10,000 times using bootstrapping with a 0.2 holdout ratio. Each iteration led to a set of selected features, of which the features we chose were common in more than 95% of the 10,000 sets as stable features related to the metastasis status.

The prognostic power of the selected features was assessed using three classifiers to distinguish between the recurrence status: (1) regularized logistic classifier, (2) SVM with a linear kernel, and (3) RF using 50 decision trees [8,9,19]. The trained classifiers were tested with the holdout set (holdout ratio of 0.2). The performance of the classifiers was assessed by the accuracy, sensitivity, and specificity of the AUC of the receiver operating characteristic (ROC) curve and *p*-value. The mean values from 10,000 bootstrapping were reported. Finally, multivariate logistic regression was conducted to assess the importance of each prognostic feature by evaluating the OR of the logistic model.

The RRS was built using a multivariate Cox’s proportional hazard model with four radiomic signatures. In this step, the original discovery cohort was used without oversampling. The RRS of each patient was defined as the relative risk of the multivariate Cox regression model, and we applied Kaplan–Meier analysis using the RRS. The discovery and validation cohorts were divided into two groups based on the optimal cutoff from the grid search. The HR and *p*-value of the log-rank test were used to measure the difference in low- and high-risk groups. Validation was performed in the same manner as the MFS analysis in the discovery cohort, except that the Cox model from the discovery cohort was refitted with a new cutoff and weights.

### 4.5. Determination of Radiomics-Feature-Associated Genes

All patients provided written informed consent for the use of their RCC tissues for genome sequencing (IRB No.: 2010-04-004). Among the discovery cohort, only 11 RCC patients had good-quality frozen tissue samples available for the WTS. RNA was extracted, quantified, quality assessed and subjected to RNA-Seq. RNA-Seq libraries were prepared using the Illumina TruSeq RNA Sample Prep kit. Sequenced reads were mapped onto hg19 using GSNAP. The initial alignment BAM files were sorted and summarized into BED files using SAMtools and bedTools. The BED files were used to calculate values of reads per kilobase of transcript per million reads (RPKM) for each gene, using the DEGseq package. This genomic discovery cohort was used to identify molecular correlates and to build a gene-expression-based surrogate of the selected CT imaging features. We identified the sets of transcripts that significantly correlated with each imaging trait (hereafter referred to as trait-associated genes) through the calculation of the Spearman correlation coefficient. The degree of overlap between signature gene sets was calculated by the Jaccard similarity coefficient, which was written as follows, where $S_{i}$ and $S_{j}$ correspond to the i-th set and the j-th set, respectively:

*J* (*S_i_*, *S_j_*) = |*S_i_* ∩ *S_j_*|/|*S_i_* ∪ *S_j_*| (3)

To verify the clinical relevance of the proposed trait-associated genes, the following information in the TCGA-KIRC dataset for RNA-Seq (*n* = 436) was downloaded from Broad GDAC Firehose (http://firebrowse.org/): (1) RCC with no metastasis (M0) and (2) the availability of survival state and OS information (*n* = 436) (Table S7).

### 4.6. Functional Analysis of Pathways and Networks, Tumor Immune Microenvironments, and the Predicted Drug Response of Trait-Associated Genes

Distinct subgroups of samples were determined by clustering hierarchically gene set ESs and cutting the tree into *k* clusters. Significant pathways (adjusted *p*-value <0.05) enriched in signature sets were identified by the pathway analysis of ReactomeFIViz, Reactome cytoscape plugin [66]. Further, functional enrichment analysis of gene–gene interaction networks was performed to identify and annotate functional gene modules. The enrichment test of each trait-associated genes composed of the top-ranked 100 genes was performed using gene set variation analysis (GSVA) [67]. Fractions of immune-associated cell types were calculated by CIBERSORT using WTS data [68]. The sensitivity of cancer cells to anticancer drugs using the CCLE dataset was identified by a tool for discovering drug sensitivity and gene expression associations [69].

### 4.7. Statistical Analysis

To explore the association between radiomics features and survival, Kaplan–Meier analysis was conducted in a training and validation phase. Kaplan–Meier curves between subgroups were generated using the “ggsurvplot” function in the “survminer” R package (https://cran.r-project.org/package=survminer). The HR and *p*-value of the log-rank test were used to measure the difference in low- and high-risk groups. The survival analysis of multigenes in the genomic validation cohort was performed using SurvExpress tool [70]. Analyses were performed using R (http://www.r-project.org/) and Matlab (The MathWorks, Natick, MA, USA).

### 4.8. Data Access

Data and materials availability: The sequencing data generated in this study have been submitted to the NCBI’s GEO (accession No. GSE135091).

## 5. Conclusions

The radiogenomics analysis of pT1 RCC in the present study showed multiple associations between semantic image features and trait-associated genes. Our radiomics signatures and trait-associated gene set could aid more effective and rationale-driven adjuvant immunotherapeutic strategies in the case of pT1 RCC demonstrating postoperative metastasis. Although we focused on RCC, the strength of our results is encouraging. Herein, radiogenomics based on CT could be used to successfully predict clinical outcomes in clinically heterogeneous pT1 RCC.

## Supplementary Materials

The following are available online at https://www.mdpi.com/2072-6694/12/4/866/s1, Table S1: Patient demographics and clinical information, Table S2: Patient demographics and clinical information in the radiomics validation cohort, Table S3: Trait-associated gene list, Table S4: List of genes using in geneset enrichment analysis, Table S5: Pathway enrichment of trait-associated genes, Table S6: Survival significance of genes in pathways, Table S7: Gene-set enrichment scores and inferred relative proportion of immune cell types of TCGA-KIRC RNA-seq gene expression profile.
